# Lateralized frontal activity for Japanese phonological processing during child development

**DOI:** 10.3389/fnhum.2015.00417

**Published:** 2015-07-17

**Authors:** Takaaki Goto, Yosuke Kita, Kota Suzuki, Toshihide Koike, Masumi Inagaki

**Affiliations:** ^1^Department of Developmental Disorders, National Institute of Mental Health, National Center of Neurology and Psychiatry, TokyoJapan; ^2^Department of Education, Tokoha University, ShizuokaJapan; ^3^Department of Education, Tokyo Gakugei University, TokyoJapan

**Keywords:** phonological awareness, development, dorsolateral prefrontal cortex, near-infrared spectroscopy, Japanese language

## Abstract

Phonological awareness is essential for reading, and is common to all language systems, including alphabetic languages and Japanese. This cognitive factor develops during childhood, and is thought to be associated with shifts in brain activity. However, the nature of this neurobiological developmental shift is unclear for speakers of Japanese, which is not an alphabetical language. The present study aimed to reveal a shift in brain functions for processing phonological information in native-born Japanese children. We conducted a phonological awareness task and examined hemodynamic activity in 103 children aged 7–12 years. While younger children made mistakes and needed more time to sort phonological information in reverse order, older children completed the task quickly and accurately. Additionally, younger children exhibited increased activity in the bilateral dorsolateral prefrontal cortex (DLPFC), which may be evidence of immature phonological processing skills. Older children exhibited dominant activity in the left compared with the right DLPFC, suggesting that they had already acquired phonological processing skills. We also found significant effects of age and lateralized activity on behavioral performance. During earlier stages of development, the degree of left lateralization appears to have a smaller effect on behavioral performance. Conversely, in later stages of development, the degree of left lateralization appears to have a stronger influence on behavioral performance. These initial findings regarding a neurobiological developmental shift in Japanese speakers suggest that common brain regions play a critical role in the development of phonological processing skills among different languages systems, such as Japanese and alphabetical languages.

## Introduction

The ability to read is vital to modern life. The action of reading words requires several abilities, including phonological awareness, vocabulary, naming speed, and visual perception (e.g., Cunningham et al., 1990; Carver, 1992). Of these, phonological awareness is an important predictor of reading performance in the late stage of child development (Liberman et al., 1974). Phonological awareness refers to the ability to detect phonological structures in spoken or mentally recalled sounds and to discriminate between these and/or minimal units of the phoneme (Yopp, 1992; Torgesen et al., 1997). This awareness normally arises in the early stage of child development and continues to improve gradually during childhood. Deficits in phonological awareness are often seen in children with developmental dyslexia who have severe difficulties reading and writing (Liberman, 1973; Chiappe et al., 2001; Ramus, 2001). Atypical brain functions are thought to underlie such deficits in this population (Paulesu et al., 2001; Temple et al., 2001; Shaywitz et al., 2002).

Several brain regions are thought to play a role in the processing of phonological information, which are revealed by recent neurophysiological studies using functional magnetic resonance imaging (fMRI) and electroencephalogram (EEG), (e.g., Bitan et al., 2007; Khateb et al., 2007; Kovelman et al., 2012). These include the left inferior frontal gyrus, superior temporal gyrus, left temporoparietal lobe, and fusiform gyrus (Temple et al., 2001; Shaywitz et al., 2002; Hoeft et al., 2006). While previous studies have reported on the involvement of these regions, the specific nature of this activity appears to be dependent on the component of the phonological process. For example, the left angular gyrus performs grapheme-to-phoneme transformations (Bitan et al., 2007), while the left superior temporal gyrus is implicated in constructing phonological representations from serial auditory information (Buchsbaum et al., 2001). The left dorsolateral prefrontal cortex (DLPFC), including the inferior frontal gyrus, stores articulatory representations (Zatorre et al., 1992, 1996) and is thought to play a critical role in the development of phonological awareness (Booth et al., 2004). The left DLPFC shows hyperactivity during phonological awareness tasks, even in very young children (e.g., ~5 years of age, Kovelman et al., 2012) and the intensity of left DLPFC activity is related to phonological processing skills during childhood (Turkeltaub et al., 2003). The above findings are mostly taken from studies of alphabetic languages. Thus, it is not clear whether the same brain functions are involved in the development of phonological awareness in the Japanese linguistic system, which is markedly different from that of alphabetical languages. Of these regions, the left DLPFC needs first to be considered in Japanese children because this region is responsible for development of phonological awareness (Turkeltaub et al., 2003; Booth et al., 2004; Kovelman et al., 2012). However, DLPFC also contributes to several cognitive functions such as working memory, set-shifting, and inhibition (Barbey et al., 2013; Forbes et al., 2014) so that we have to rule out other cognitive functions except for phonological awareness when we measure activity of DLPFC. Here, we introduce experimental design of cognitive subtraction. For one instance, we subtract brain activity on tasks for phonological storing (i.e., baseline task) from the activity on other tasks for phonological storing and manipulations (i.e., experimental task), which enables us to extract the activity for phonological manipulation and to minimize the effect of other cognitive functions. Although this design has several limitations such as ignorance of interaction effects (e.g., Friston et al., 1996) and we should interpret results carefully, we can minimize number of experimental conditions and evaluate brain activity in young children without excessive mental and/or cognitive stresses.

The Japanese language has a unique linguistic system with two kinds of characters, kana and kanji. While kanji (i.e., Chinese characters) are ideograms, kana are phonograms which serve as a base for fundamental characters in Japanese. The phonological units represented by kana are moras, which usually consist of a single vowel with/without a single consonant (V or CV). Kana has an extremely straightforward correspondence between graphemes and phonological units, such that a single kana character denotes a single syllable (i.e., mora, Wydell and Butterworth, 1999). In native Japanese speakers, phonological awareness develops in early childhood and becomes refined during middle childhood (Hara, 2001). A neuroimaging study of Japanese adults revealed that brain activation during phonological processing is dependent on the stimulus modality, for example, the bilateral superior temporal sulci activate in response to auditory stimuli and the bilateral temporoparietal lobes activate in response to visual stimuli (Seki et al., 2004). However, few studies have focused on the development of neural mechanisms underlying phonological awareness in Japanese children. Thus, it is unclear whether processing of phonological information in Japanese activates the same brain regions as for alphabetic languages during childhood.

The present study aimed to reveal developmental changes in the neural activity underlying phonological processing in Japanese children. Previous studies have used several phonological processing tasks such as rhyming, phoneme identification, segmentation, blending, and manipulation (Yopp, 1992, 1995; Stahl and Murray, 1994). We adopted the mora reversal task (Seki et al., 2008), which takes the specific characteristics of the Japanese language into account. The task requires participants to listen to one word and then to say the morae of the word in reverse order, meaning that the task requires a high level of phonological processing skill. We expected that the demanding nature of the task would be ideal for revealing developmental changes in ability and corresponding neural changes. We used near-infrared spectroscopy (NIRS) to measure brain activity in Japanese children during the task, as this technique is suitable for both young participants (Kita et al., 2011; Wigal et al., 2012; Kikuchi et al., 2013) and auditory experiments because of minimal noise (unlike other techniques such as fMRI). NIRS is also less affected by movement artifact (Kita et al., 2011) and useful for measuring brain activity during the tasks in which the subjects are required to respond orally because young children tend to move when they speak. Additionally, we can place NIRS probes on children’s heads so easily and quickly that we can sample large number of children and reveal developmental shifts based on the reliable sample size. Given our task and measuring system, we hypothesized that the left DLPFC would play an important role in phonological processing in Japanese children, similar to that seen for alphabetic languages.

## Materials and Methods

### Participants

A total of 103 right-handed native Japanese children (age range = 7.0–12.8 years, 52 females and 51 males) from a local public school were paid for their participation. We placed them into three age groups (Low, 7–8 years: Middle, 9–10 years: High, 11–12 years). We assessed verbal and non-verbal intellectual abilities using the number recall test (Kaufman Assessment Battery for Children, Matsubara et al., 1993) and Raven’s Colored Progressive Matrices Test (Raven, 1976). The scores produced by the children in the three groups were within the normal range and consistent within the groups (**Table 1**). All participants were free from neurological and psychiatric disorders, according to reports from their parents. Written informed consent was obtained from all participants and their parents prior to the experiments. The research protocol was approved by the ethics committee at the National Center of Neurology and Psychiatry (approval number 20-8-JI10).

**Table 1:** Participant characteristics.

Group	Low	Middle	High	*P-*value
*N*	42	35	26	
(Male : Female)	(19:23)	(20:15)	(13:13)	0.20
Age (month)	95.0 ± 7.0	117.4 ± 6.6	141.4 ± 7.1	
	(84–106)	(108–129)	(132–153)	
RCPM	30.7 ± 2.5	30.5 ± 3.5	31.6 ± 2.0	0.29
	(26–35)	(21–35)	(26–36)	
K-ABC				
Number recall (SS)	11.3 ± 2.6	11.8 ± 2.0	12.3 ± 2.8	0.31
	(7–19)	(7–16)	(7–19)	

*Participant characteristics with means ± SD (range). These variables were compared among the three groups using a chi-square test for the sex ratio, and one-way analyses of variance (ANOVA) for Raven’s Coloured Progressive Matrices (RCPM) and for Kaufman Assessment Battery for Children (K-ABC) number recall Scaled scores (SS).*

### Stimuli and Tasks

For auditory word stimuli we used 20 concrete nouns with a length of three morae (about 1 s). The nouns had degrees of imaginability greater than 5.8 out of 7.0 (Tokyo Metropolitan Institute of Gerontology and NTT Communication Science Laboratories, 2005), indicating that Japanese children could easily understand all of the words. We digitally recorded the stimuli, which were spoken by a female native Japanese speaker. The peak of the sound intensity was equalized across stimuli using Sound It! Basic for Windows (Internet Co. Ltd. Osaka, Japan; total root mean squares ranged between -10.82 and -15.52 dB).

**FIGURE 1:**
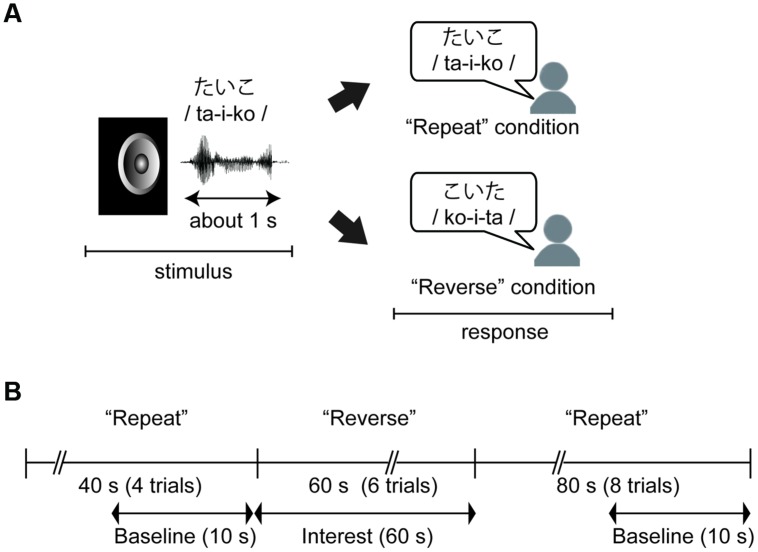
**(A)** An example of the behavioral task. Children were required to simply repeat the stimulus in the “Repeat” condition, and to repeat a series of morae corresponding to the stimulus in the “Reverse” condition. **(B)** Time course of the task and near-infrared spectroscopy (NIRS) measurements. The task was divided into three sections: Repeat section (0–40 s: four trials), Reverse section (40–100 s: six trials), and Repeat section (100–160 s). NIRS measurements were performed throughout the three sections, and these data were analyzed with baseline corrections from two baseline data periods to extract hemodynamic activity for phonological processing.

We conducted a mora reversal task (see, **Figure 1A**) using C++ Builder XE2 (Embarcadero Technologies Inc. San Francisco, CA, USA). In the task, each participant was asked to respond aloud to a stimulus in either a “Repeat” or “Reverse” condition. In the “Repeat” condition, the participant was required to simply repeat the stimulus (e.g., ta-i-ko to ta-i-ko), and in the “Reverse” condition, the participant was instructed to repeat the series of morae corresponding to the stimulus in reverse order (e.g., ta-i-ko to ko-i-ta). As noted in “Introduction” Section, we used cognitive subtraction with these two conditions and this experimental contrasts in these two conditions enabled us to assess brain activity associated with phonological manipulation. Additionally, we were able to remove irrelevant factors, such as oral responses or simple auditory perception, by setting the “Repeat” condition as the baseline.

The stimuli were presented via the speaker of a laptop computer at about 60 db with a stimulus onset asynchrony (SOA) of 10 s. There were 18 trials in total, and the first four and the last eight trials were performed under the “Repeat” condition (“Repeat” section) while the remaining trials were performed under the “Reverse” condition (“Reverse” section, **Figure 1B**). Before the task, the participants completed practice trials for the “Repeat” and “Reverse” conditions using two of the stimuli. The other 18 stimuli were presented in random order during the task. Participants were informed of whether a given trial was a “Repeat” or “Reverse” trial by a visual word cue presented on the screen for 2 s before the first trial in each section.

### Measurements of Behavioral Data

Behavioral data were recorded using an IC recorder (HM-200, Sanyo Inc., Osaka, Japan). Response times (RTs) were defined as the duration between the stimuli onset and the end of the response. The offsets of the responses were identified using Sound It! Basic 6.0 for Windows (Internet Co. Ltd. Osaka, Japan). We performed a univariate analysis of variance (ANOVA) with Sheffer’s multiple-comparison for groups (Low, Middle, High) and conditions (Repeat, Reverse) in the mean correct RT. The ANOVA was conducted on the number of correct responses for groups.

### Measurements with Near-Infrared Spectroscopy (NIRS)

#### Recordings

**FIGURE 2:**
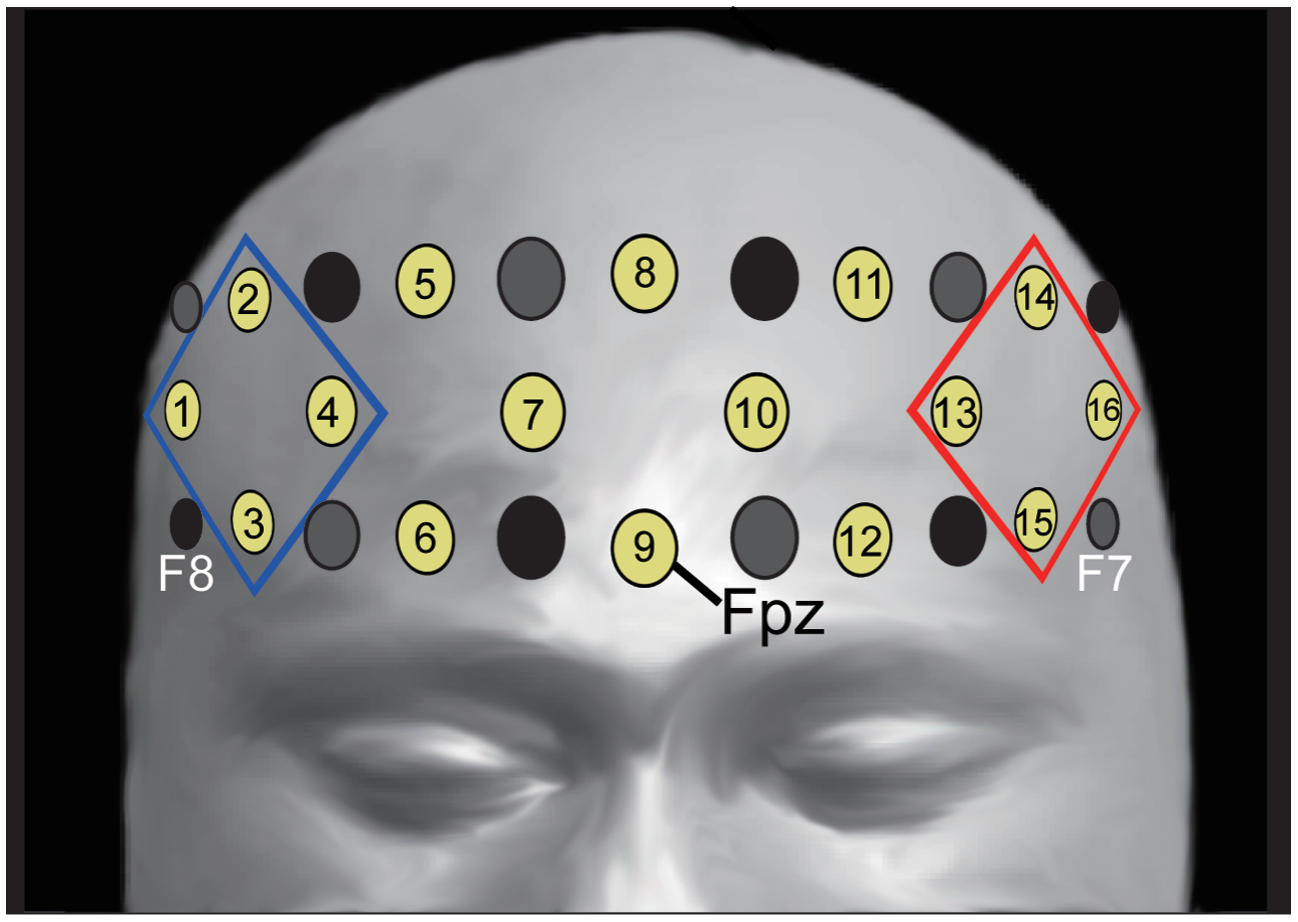
**Location of emitter (gray) and detector (black) probes and channels.** Channel 9 was placed on Fpz (midpoint between Fp1 and Fp2), the probe at the bottom left corner was placed around F7, and the probe at the bottom right corner was placed around F8, in accordance with the international 10–10 system (for more information see Kita et al., 2011; Tsujimoto et al., 2013). We analyzed the averaged oxy-Hb signals from the channels at the left and right frontal areas (R-ROI: 1, 2, 3, 4 ch, blue rhombus, L-ROI: 13, 14, 15, 16 ch, red rhombus).

We recorded changes in the concentration of oxygenated hemoglobin (oxy-Hb) at 16 locations on the forehead with a temporal resolution of 650 ms (OEG-16 Spectratech Inc., Yokohama, Japan). In our system, near-infrared laser diodes (emitter probes) emitted two wavelengths (~770 and 840 nm) and the reemitted lights were detected on avalanche photodiodes (detector probes) located 3.0 cm apart from each emitter probe (Kita et al., 2011; Tsujimoto et al., 2013). Six emitter and detector probes were arranged in a 6 × 2 matrix. The center point of the bottom of the matrix was placed at Fpz, and the left and right corners of the bottom were located approximately at F7 and F8, respectively, in accordance with the international 10–10 system (**Figure 2**). Hence, we were able to obtain data from 16 locations between the emitter and detector probes.

#### Filtering

The NIRS data were low pass filtered oﬄine at 0.05 Hz using a fast Fourier transform (FFT) to remove artifacts caused by minor movements of the participant (Kita et al., 2011; Makizako et al., 2013) because the NIRS data in the present setting was almost sustained across a block (i.e., 60 s) and was not synchronized to each stimulus (**Supplementary Figure S1**). We then carried out independent component analysis (ICA) for additional artifact rejection.

Independent component analysis has been reported to be helpful in removing artifacts from physiological data [e.g., electroencephalography (EEG), Delorme et al., 2007]. Using ICA, data can be decomposed into statistically independent components that are linearly related to the original data, and after analysis and removal of the artifacts, the original data can be linearly restored from the components. If all artificial components are excluded, the restored data become artifact-free. Recently, ICA has been applied to NIRS data (Kohno et al., 2007; Medvedev et al., 2008).

The changes in oxy-Hb measured by NIRS can be contaminated by the skin blood flow (Takahashi et al., 2011). Skin blood flow is easily affected by activity in the automatic nervous system, in which spatial distribution is not localized because of zone of autonomic innervation (Kohno et al., 2007). Conversely, we considered activity in the frontal area to be localized during phonological manipulation (Kovelman et al., 2012). Hence, we were able to discriminate between the components associated with skin blood flow and cortical activation based on the spatial distribution. In this study, NIRS data were decomposed to 16 components using the FastICA R package (Marchini et al., 2007), and components associated with skin blood flow, i.e., characterized by an overall increase across all channels (Kohno et al., 2007), were excluded by visual inspection. The number of excluded components was 1–3 component(s) in each participant. We used the restored data for further analysis.

#### Analysis

We used linear fitting to make baseline corrections based on two baseline intervals: the mean across the 10-s-period before the “Reverse” section, and the mean across the final 10-s-period of the second “Repeat” section (**Figure 1B**). To assess the laterality of activity during the “Reverse” section, we selected the averaged values in the channels corresponding to the right- and left- DLPFC as the regions of interest [right region of interest (R-ROI): 1-, 2-, 3-, and 4-ch; left region of interest (L-ROI): 13-, 14-, 15-, and 16-ch, **Figure 2**]. Moreover, we defined the L–R index as the averaged value of L-ROI minus that of R-ROI to simply assess the degree to laterality. For the averaged ROI values, we conducted a two-way mixed factorial ANOVA with Sheffer’s multiple-comparison including groups (Low, Middle, High) and locations (R-ROI, L-ROI).

We assumed that developmental trajectory of left-lateralization is different among individuals, and the individual difference influenced the maturation of phonological manipulation. Hence, the hierarchical regression analysis (Cohen et al., 2003) was conducted on mean correct RT including age, L–R index, and an interaction between these variable as predictors. All independent variables were centered on their means. In the first step, age (month) and the L–R index were entered in the model. In the second step, the interaction between age and the L–R index was added. When the interaction was significant, we investigated further using simple slope analyses: slopes for the regression analyses were computed at 1 SD above and below the mean (Aiken and West, 1991). Data processing and statistical analyses were performed with R software (R Development Core Team, 2005) and SPSS 19.0 (SPSS. Japan Inc., Tokyo, Japan).

## Results

### Behavioral Results

The mean correct RTs and the number of correct responses in each age group are illustrated in **Table 2**. Regarding to mean correct RTs, we found significant main effect of groups and conditions, *F*(2,100) = 7.65, *p* < 0.001; *F*(1,100) = 107.34, *p* < 0.01 and a significant interaction between these variables, *F*(2,100) = 9.15, *p* < 0.001. A simple effect analysis revealed that a main effect of groups was found in Reverse section, where the mean correct RT was longer in the Low compared with the Middle group and we found the shortest RT in the High group (*p* < 0.05). On the other hand, there was no significant effect of group in Repeat section.

**Table 2:** Behavioral performance.

		Group			Comparison among groups
		Low	Middle	High	*Post hoc*
Response time (ms)	Repeat	1817 ± 204	1926 ± 255	1860 ± 245	Low = Middle = High
	Reverse	3810 ± 1437	3203 ± 1066	2529 ± 1118	Low > Middle > High
Correct response (number)	Repeat	12	12	12	Low = Middle = High
	Reverse	4.97 ± 1.38	5.63 ± 0.77	5.80 ± 0.49	Low < Middle = High

*Behavioral performance with mean scores ± SD. These variables were compared using a one-way analysis of variance (ANOVA) with Bonferroni’s multiple-comparisons.*

In terms of the number of correct response all participants correctly respond for each stimulus in Repeat section (*n* = 12, i.e., accuracy = 100%). In the Reverse section, ANOVA showed a significant main effect of groups, *F*(2,100) = 6.51, *p* = 0.002. *Post hoc* tests revealed that the number of correct responses was larger in the High and Middle groups than in the Low group (*p* < 0.05).

### NIRS Results

**FIGURE 3:**
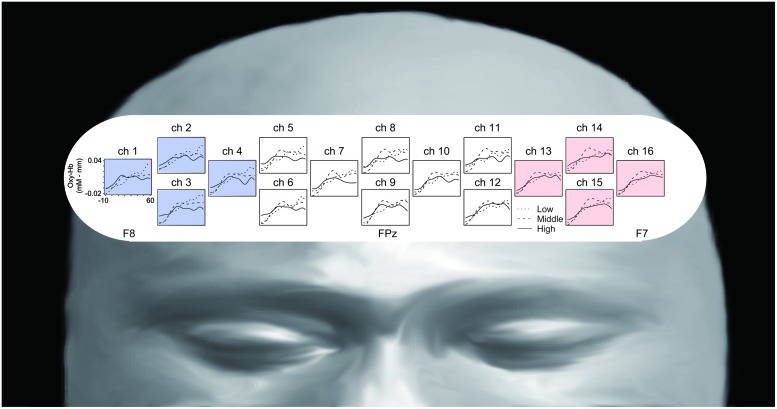
**Grand averaged waveforms of all channels for the three groups, generated using a 70 s period starting 10 s before the “Reverse” section**.

**FIGURE 4:**
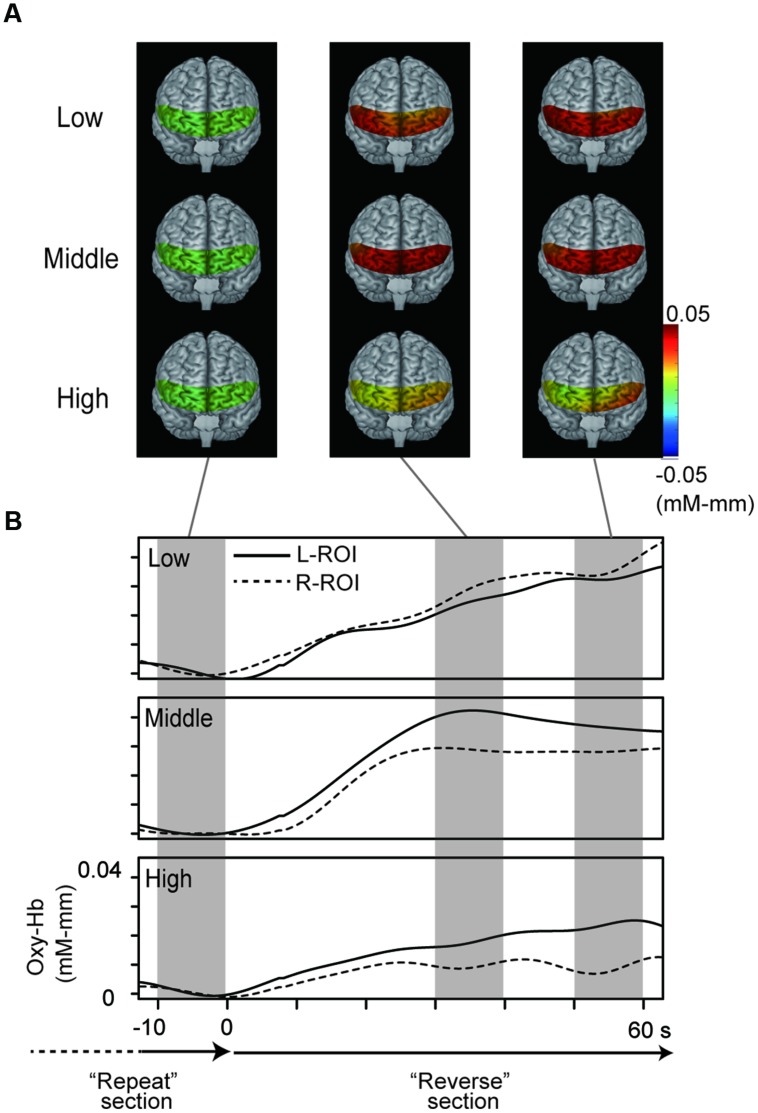
**NIRS data from the three groups. (A)** Topographies of hemodynamic activity constructed using averaged data for the three groups. **(B)** Grand averaged waveforms for the three groups at each ROI, generated using a 70 s period starting 10 s before the “Reverse” section.

**Figure 3** represents oxy-Hb waveforms of all channels for the 70 s period starting 10 s before the “Reverse” section. **Figure 4** shows the oxy-Hb maps and the oxy-HB waveforms at L-ROI and R-ROI. We observed left lateralized activation in the Middle and High groups but not in the Low group. In terms of the waveforms of the ROIs, we found that the increase in oxy-Hb signals was larger at the L-ROI than the R-ROI in the Middle and High groups.

**FIGURE 5:**
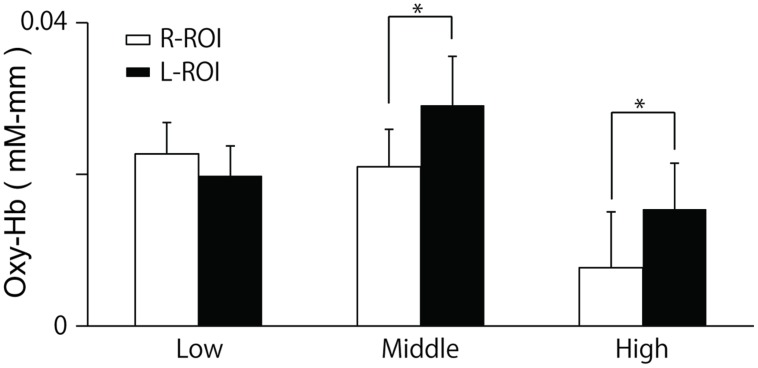
**Means and SE of oxy-Hb signals during last 10 s of the ‘Reverse’ section at each ROI.** The R-ROI and L-ROI were set to correspond to the right DLPFC and left DLPFC, respectively. *p < 0.05.

The two-way ANOVA revealed a significant interaction between group and location, *F*(2,100) = 5.26, *p* = 0.01. We did not find a difference between the oxy-Hb signals in the L-ROI and R-ROI in the Low group, *F*(1,41) = 2.54, *p* = 0.12, whereas the signals were significantly larger at the L-ROI than the R-ROI in the Middle and High groups, *F*(1,34) = 5.88, *p* = 0.02, *F*(1,25) = 4.36, *p* = 0.04 (**Figure 5**). We found no significant main effects.

To examine the possibility that the error-related activity contaminated previous results, we excluded 18 participants (Low: *n* = 11, Middle: *n* = 4, High: *n* = 3) and performed statistical analysis. Consistent with the previous analysis, the two-way ANOVA revealed a significant interaction between group and location [*F*(2,82) = 4.28, *p* < 0.05]. We also revealed the significant differences between L-ROI and R-ROI in Middle and High groups (*p* < 0.05) and no significant difference in Low groups (*p* = 0.26). In addition, we further performed a two-way mixed factorial ANOVA including accuracy (participants with all corrects, ones with some errors) and locations (R-ROI, L-ROI) in Low groups. There was no significant main effect of accuracy [*F*(1,40) = 0.02, *p* = 0.90]. These results indicated that number of error trials did not affect the NIRS results.

### Hierarchical Multiple Regression Analysis

The results of the hierarchical multiple regression analyses are shown in **Table 3**. The model was significant in the first step, *F*(2,100) = 11.08, *p* < 0.001. In the second step, the addition of the interaction between the L–R index and age improved predictive power, *F*(3,99) = 10.19, *p* < 0.001. Interestingly, we found a significant interaction between age and the L–R index in the second step (β = -0.24, *p* = 0.009). Simple slopes analyses revealed that age significantly predicted the mean correct RT at 1 SD above the mean of the L–R index (β=-0.74, *p* < 0.001) and age did not significantly predict the mean correct RT at 1 SD below the mean of the L–R index (β=-0.18, *p* = 0.179). These results indicate that children with left-lateralized brain activity tend to show improvements in behavioral performance as they age, while this improvement is less likely in children with non-lateralized activity.

**Table 3:** Hierarchical multiple regression analysis predicting behavioral performance.

	Step	
	1	2
Independent variables	β	β
Age (months)	-0.44**	-0.46**
L–R index	-0.10^ns^	-0.15^ns^
Age × L–R index		-0.24**
*R*^2^	-0.18**	-0.24**
Δ*R*^2^	-0.18**	-0.06**
Adj *R*^2^	-0.17**	-0.21**

ns, not significant; **p < 0.01.

## Discussion

In the present study, we conducted the mora reversal task with native-born Japanese children aged 7–12. We examined developmental shifts in brain activity associated with phonological processing using the NIRS system. This system is well suited for neuroimaging with an auditory-vocal experimental paradigm and a large sample population of children. While younger children made more mistakes and needed more time to sort phonological information in reverse order, older children completed the task quickly and accurately. Neuroimaging data revealed the children in the Middle and High groups to have increased brain activity in the left compared with the right DLPFC during the task, although this was not observed in the children in the Low group. Additionally, we found significant effects of age and lateralized activity on behavioral performance. During the early stage of development, the degree of left lateralization had a smaller effect on behavioral performance. However, the degree of left lateralization had a stronger influence on behavioral performance in the late stage of development. Our findings suggest that a common brain region plays a critical role in the development of phonological processing among different languages systems, including Japanese and alphabetic languages.

We found that behavioral performance on the mora reversal task gradually improved with age, as indicated by decreasing RTs and rising accuracy. This indicates that phonological awareness continues to grow until at least age 12 in Japanese speakers. The phonological awareness task is thought to be hierarchical, in that it can measure behavior on multiple levels, from easy to difficult (Treiman and Zukowski, 1991; Yopp and Yopp, 2000). Children in early childhood may only be able to engage in rhyming or identification tasks (the lower tier of the hierarchy), but they gradually grow and acquire skills until they can complete more difficult tasks, such as blending, deletion, and manipulating. The reversal task in the present study is located high in the hierarchy of difficulty because it requires the child to segment phonological information from an auditory stimulus, manipulate the information by reversing the order, and finally blend the information together. Using this higher-level task, we were able to clarify the maturational development of phonological awareness in Japanese children, and use the behavioral evidence to examine developmental shifts in brain activity underlying phonological processing.

We found that developmental changes in brain activity, as indicated by activation during the mora reversal task, became more left-lateralized as the development of phonological awareness increased. Previous studies have reported developmental shifts in brain activity for cognitive tasks like verbal fluency, specifically, the distributions range from diffuse to focal as children mature (Gaillard et al., 2000; Durston et al., 2006). The maturation of cognitive ability appears to be characterized by a diminishing of irrelevant brain activity while relevant activity remains. The left DLPFC, the focal region identified in the present study, plays a pivotal role in phonological awareness from the early stages of the development (Kovelman et al., 2012) and the degree of activity in the left DLPFC has been correlated with phonological processing skills during childhood (Turkeltaub et al., 2003). As these associations are evident only on the left side, we consider left-lateralized brain activity in the present task to reflect a maturational pattern of brain activity underlying the development of phonological awareness in Japanese speakers.

It appears that left-lateralization of brain activity affects behavioral performance differently depending on a child’s age. Specifically, the influence of left-lateralization is blurred in younger children, while it is clearly apparent in older children. This developmental shift of the influence of left-lateralization is thought to be closely linked to the linguistic characteristics of the Japanese language. In Japanese kana, there is a direct correspondence between phonology and orthography, which means that one character strictly corresponds to one syllable (Wydell and Butterworth, 1999; Seki et al., 2004). This correspondence enables easy back-and-forth transformations between phonemes and graphemes. Thus, it is possible that Japanese speakers can easily access both auditory and visual information even if phonological tasks are conducted with only auditory stimuli. Additionally, some children may have used a mental representation of a kana location table while completing the present task. A kana location table is used for language learning, and consists of kana characters placed in a grid with 5 (vowels) by 10 (consonants). It is easy to locate the kana characters on the table, and auditory information can be transformed into visual information rapidly and accurately. Young Japanese children are especially familiar with the kana location table because it is often used for acquisition of kana in early elementary grades. Seki et al. (2004) reported that some Japanese participants used the table during a phonological task. It is possible that with the advantages of direct correspondence and the location table, some participants may have been able to perform the present task using both auditory (i.e., phoneme) and visuospatial information (i.e., grapheme) despite being unable to complete the task with only phonological information.

Previous neuroimaging studies have revealed that the right DLPFC, including the inferior frontal cortex, is involved in visuospatial processing (McCarthy et al., 1996; Owen et al., 1998; Smith and Jonides, 1999). Hoshi et al. (2000) also reported that right DLPFC are active when the normal participants transformed auditory information into visuospatial information in the backward digit span task, which is similar to the present task. In the present study, the younger participants may have used visuospatial in addition to phonological information, and thus exhibited activation in the bilateral DLPFC. In contrast, older participants may have relied only on phonological information to accomplish the task, and thus did activate the right DLPFC only to a lesser extent than left DLPFC. This lateralization appears to reflect a neurobiological change underlying the development of phonological processing skills specific to the Japanese language.

In the present study, we used the NIRS system to examine neurobiological characteristics in a population of young children who generally are not comfortable in restrictive environments such as a MRI scanner. Despite this advantage, the NIRS system has some technical limitations. We encountered difficulty when attempting to measure brain activity in deep areas involved in phonological function, such as the basal ganglia (Kita et al., 2013) because the system employs near infrared lights, and is thus suitable for measuring activity in cortex only. In addition, activity at ROIs is not only associated with activity of DLPFC and the inferior frontal cortex. In one study with adults, activities of these regions were evaluated independently (Tupak et al., 2012), whereas we could not discriminate these regions because of head size of children. Since left DLPFC has a pivotal role of the phonological manipulation (Kovelman et al., 2012), we considered that our NIRS results were mainly associated with left DLPFC rather than left IFG. We also focused only on the frontal area, as it is a critical brain region for phonological processing, and did not discuss connectivity between the DLPFC and other areas. These technical limitations could be addressed in future research using NIRS systems with more channels than the one used in our study (e.g., Pu et al., 2013). Another limitation is experimental design. While we introduced cognitive subtraction design for extracting brain activity for phonological manipulation in young children, we cannot entirely exclude the effects of other cognitive functions. The present NIRS data, which were acquired by subtracting the activities on “Repeat” condition from those on “Reverse” condition, might include interaction effects of both conditions (e.g., Friston et al., 1996) and we should interpret the present results carefully. Future research could employ fMRI with careful experimental design such as factorial design, considering both participant age (i.e., young children), and task characteristics (i.e., auditory stimuli and oral response). This may reveal more detail regarding the neurobiological changes underlying phonological processing in Japanese speakers. We could not conclude the relationship between left-lateralized brain activity and the maturation of phonological manipulation because of cross-sectional data. Especially, the present study has difficulty to specify a direction of causal relationships between behavioral performance and lateralized brain activity varied with age. Further study is expected with longitudinal data of Japanese population to confirm the present results. We should also, using sophisticated experimental settings, examine whether or not the children show different brain activity when they do not have any differences of behavioral performance, which helps us specify the direction of the causal relationships.

## Conclusion

We measured hemodynamic activity in a population of Japanese children, and observed a neurobiological change during the course of development. Younger children had increased activity in the bilateral DLPFC, which may reflect immature phonological processing skills. Conversely, older children showed dominant activity in the left DLPFC compared with the right DLPFC, which suggests that they had already acquired phonological processing skills. Thus, it appears that brain activity in the frontal area is lateralized during the development of phonological processing in Japanese speakers. These initial findings are useful for discussions of the neurobiological characteristics of children with developmental dyslexia, who are too young to undergo fMRI studies. We anticipate that our results will lead to a better understanding of dyslexia in Japanese speakers.

## Conflict of Interest Statement

The authors declare that the research was conducted in the absence of any commercial or financial relationships that could be construed as a potential conflict of interest.

## Supplementary Material

Figure 1 **NIRS waveforms of different filter setting (representative data)**. Red line shows waveforms low-pass filtered at 0.5 Hz and black line shows waveforms low-pass filtered at 0.05 Hz. The peaks of the waveforms low-pass filtered at 0.5 Hz are not synchronized with stimuli (i.e., more than 1 peaks within 10 s interval), meaning that these peaks are artifactsirrelevant to the experimental tasks or stimuli. Thus, we adopt the low-pass filter at 0.05Hz.
