# Supplementary figures and images for: Lateralized frontal activity for Japanese phonological processing during child development
Source: Front Hum Neurosci. 2015 Jul 17;9:417. doi: 10.3389/fnhum.2015.00417 (PMC4505106; doi:10.3389/fnhum.2015.00417)

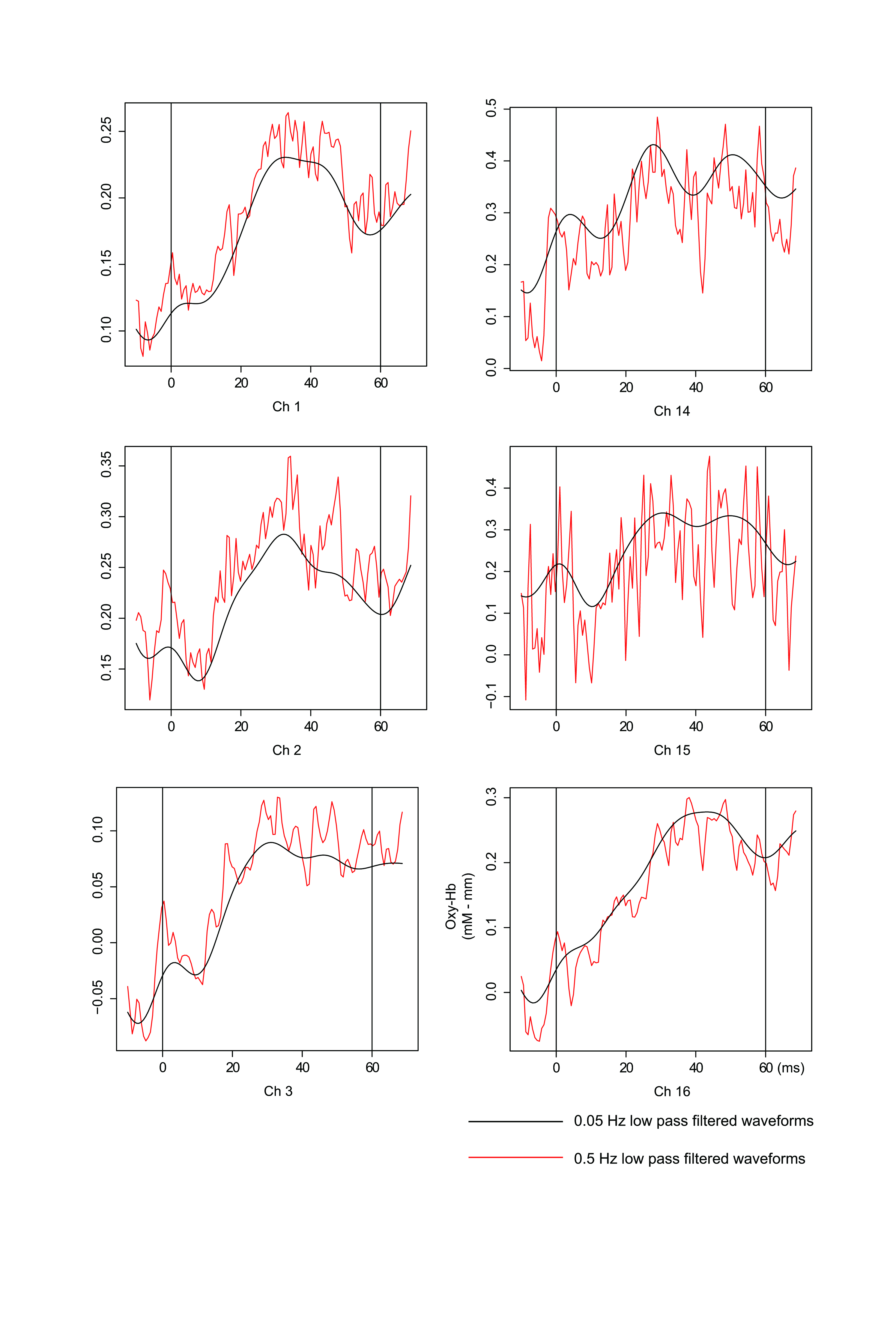

Supplement: Figure 1 — NIRS waveforms of different filter setting (representative data). Red line shows waveforms low-pass filtered at 0.5 Hz and black line shows waveforms low-pass filtered at 0.05 Hz. The peaks of the waveforms low-pass filtered at 0.5 Hz are not synchronized with stimuli (i.e., more than 1 peaks within 10 s interval), meaning that these peaks are artifactsirrelevant to the experimental tasks or stimuli. Thus, we adopt the low-pass filter at 0.05Hz. [file Image_1.TIF]
